# CAMI Benchmarking Portal: online evaluation and ranking of metagenomic software

**DOI:** 10.1093/nar/gkaf369

**Published:** 2025-05-07

**Authors:** Fernando Meyer, Gary Robertson, Zhi-Luo Deng, David Koslicki, Alexey Gurevich, Alice C McHardy

**Affiliations:** Computational Biology of Infection Research, Helmholtz Centre for Infection Research (HZI), 38124 Braunschweig, Germany; Braunschweig Integrated Centre of Systems Biology (BRICS), Technische Universität Braunschweig, 38106 Braunschweig, Germany; Initiative for the Critical Assessment of Metagenome Interpretation (CAMI ); Computational Biology of Infection Research, Helmholtz Centre for Infection Research (HZI), 38124 Braunschweig, Germany; Braunschweig Integrated Centre of Systems Biology (BRICS), Technische Universität Braunschweig, 38106 Braunschweig, Germany; Initiative for the Critical Assessment of Metagenome Interpretation (CAMI ); Computational Biology of Infection Research, Helmholtz Centre for Infection Research (HZI), 38124 Braunschweig, Germany; Braunschweig Integrated Centre of Systems Biology (BRICS), Technische Universität Braunschweig, 38106 Braunschweig, Germany; Initiative for the Critical Assessment of Metagenome Interpretation (CAMI ); Initiative for the Critical Assessment of Metagenome Interpretation (CAMI ); Computer Science and Engineering, Penn State University, University Park, PA 16802, United States; Biology, Penn State University , University Park, PA 16802, United States; Initiative for the Critical Assessment of Metagenome Interpretation (CAMI ); Helmholtz Institute for Pharmaceutical Research Saarland (HIPS), Helmholtz Centre for Infection Research (HZI), 66123 Saarbrücken, Germany; Center for Bioinformatics Saar and Saarland University, Saarland Informatics Campus, 66123 Saarbrücken, Germany; Computational Biology of Infection Research, Helmholtz Centre for Infection Research (HZI), 38124 Braunschweig, Germany; Braunschweig Integrated Centre of Systems Biology (BRICS), Technische Universität Braunschweig, 38106 Braunschweig, Germany; Initiative for the Critical Assessment of Metagenome Interpretation (CAMI ); German Center for Infection Research (DZIF), partner site Hannover Braunschweig, 38124 Braunschweig, Germany; Cluster of Excellence RESIST (EXC 2155), Hannover Medical School, 30625 Hannover, Germany

## Abstract

Finding appropriate software and parameter settings to process shotgun metagenome data is essential for meaningful metagenomic analyses. To enable objective and comprehensive benchmarking of metagenomic software, the community-led initiative for the Critical Assessment of Metagenome Interpretation (CAMI) promotes standards and best practices. Since 2015, CAMI has provided comprehensive datasets, benchmarking guidelines, and challenges. However, benchmarking had to be conducted offline, requiring substantial time and technical expertise and leading to gaps in results between challenges. We introduce the CAMI Benchmarking Portal—a central repository of CAMI resources and web server for the evaluation and ranking of metagenome assembly, binning, and taxonomic profiling software. The portal simplifies evaluation, enabling users to easily compare their results with previous and other users’ submissions through a variety of metrics and visualizations. As a demonstration, we benchmark software performance on the marine dataset of the CAMI II challenge. The portal currently hosts 28 675 results and is freely available at https://cami-challenge.org/.

## Graphical Abstract

**Graphical Abstract:**
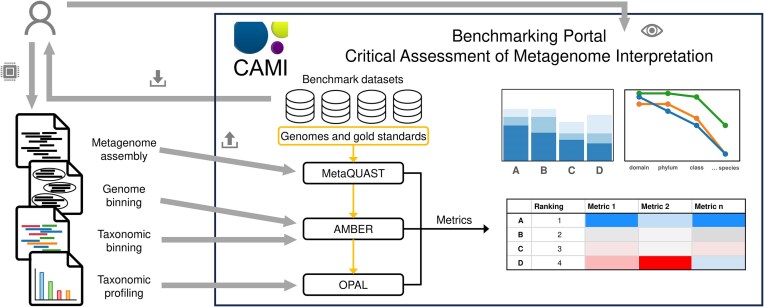

## Introduction

Metagenomics, the study of the function and structure of microbial DNA isolated and sequenced directly from environmental samples, enables a comprehensive exploration of microbial communities, with applications ranging from human health and pathogen detection [1] to agriculture [2] and even space exploration [3, 4]. It relies on numerous computational methods, making benchmarking essential for assessing the current state of the art, identifying gaps, and establishing best practices. The most common categories include assembly, binning, and profiling methods. Metagenome assemblers reconstruct longer contiguous sequences (contigs) from DNA sequence reads. Genome binners group sequences inferred to originate from the same genome, thereby reconstructing metagenome-assembled genomes (MAGs), while taxonomic binners assign them to known taxonomic groups. Taxonomic profilers further estimate the identities and abundances of microbial taxa in the sequenced community.

The community-driven initiative Critical Assessment of Metagenome Interpretation (CAMI) aims to evaluate these methods by offering realistic datasets and reproducible benchmarking challenges. To date, CAMI has organized two challenges, mobilizing over 100 contributors and participants worldwide and gathering almost 500 metagenome assembly, genome and taxon binning, and taxonomic profiling submissions across a range of datasets (Table 1) [5, 6].

**Table 1.** Overview of CAMI benchmark datasets

CAMI challenge edition	Benchmark dataset	# Samples	Sequencing technology	DOI
CAMI I	Challenge high complexity	5	SR	10.5524/100344
	Challenge medium complexity	2	SR	10.5524/100344
	Challenge low complexity	1	SR	10.5524/100344
	“Toy” high complexity	5	SR	10.5524/100344
	“Toy” medium complexity	2	SR	10.5524/100344
	“Toy” low complexity	1	SR	10.5524/100344
CAMI II	Challenge plant-associated	21	SR, PacBio, ONT	10.4126/FRL01-006425521
	Challenge marine	10	SR, PacBio	10.4126/FRL01-006425521
	Challenge strain-madness	100	SR, PacBio	10.4126/FRL01-006425521
	Challenge clinical pathogen detection	1	SR	10.4126/FRL01-006425521
	“Toy” mouse gut	64	SR, PacBio	10.4126/FRL01-006421672
	“Toy” Human Microbiome Project (urogenital, skin, airways, gastrointestinal, and oral)	49	SR, PacBio	10.4126/FRL01-006425518

CAMI benchmark datasets from the CAMI I and II challenges are available for download and online evaluation of metagenome assembly, genome and taxonomic binning, and taxonomic profiling on the CAMI Benchmarking Portal (https://cami-challenge.org/). Numbers of samples are per simulated sequencing technology. SR stands for Illumina HiSeq short reads, PacBio for Pacific Biosciences long reads, and ONT for Oxford Nanopore long reads. The clinical pathogen detection challenge dataset contains real Illumina MiSeq metagenome data. Datasets are also linked and described at https://cami-challenge.org/datasets/.

Since 2015, CAMI has provided comprehensive datasets, benchmarking guidelines, and challenges through its web portal (https://cami-challenge.org/). Over time, specialized assessment software has been developed incorporating CAMI’s established metrics, such as MetaQUAST [7] for metagenome assembly assessment, AMBER [8] for genome and taxonomic binning, and OPAL [9] for taxonomic profiling evaluation. Until now, no platform supported online evaluation and ranking of methods by integrating all of these tools and CAMI datasets, making benchmarking time-consuming and requiring substantial expertise from developers.

Here, we introduce the CAMI Benchmarking Portal, a web-based platform designed to facilitate the evaluation of metagenomic software through a user-friendly web interface. By integrating MetaQUAST, AMBER, and OPAL, the portal enables users to assess software results of common metagenomic analyses using field-established metrics. The portal simplifies benchmarking, making it faster and more reproducible, and improving the FAIRness (Findability, Accessibility, Interoperability, and Reusability) [10, 11] of computationally generated data such as assemblies, MAGs, and profiles. In addition to its evaluation service, it serves as a repository where results can be ranked and compared in various visualizations. It eliminates the need for users to install and execute other methods and evaluation software for a comprehensive assessment of individual tools. It further facilitates an interactive exploration of method results, to identify the most suitable software meeting a specific performance profile, while providing a continuously adapting evaluation framework for metagenomic analyses.

## Materials and methods

### Web server overview

#### Workflow

A typical benchmarking workflow follows these steps: (i) A user downloads CAMI benchmark data from the CAMI Benchmarking Portal, (ii) applies their favorite or own method under development on the data, and (iii) uploads the results for evaluation on the portal. As input, the portal accepts assemblies, genome or taxonomic binnings, and taxonomic profiles, in the respective formats, of a CAMI benchmark dataset. For assembly, the format is FASTA. For binning and taxonomic profiling, the CAMI community established standardized tab-separated files: binning files contain one line per sequence with its corresponding bin assignment, while profiling files list taxa and their relative abundances, for one or multiple samples (for details, see https://cami-challenge.org/file-formats). For all categories, gzipped files are supported. Upon upload, the portal recognizes and validates the format automatically. For assemblies, users then manually select on the portal the corresponding dataset and samples. Binning and profiling files must include codes identifying datasets and samples uniquely, allowing the portal to select these automatically and eliminating the need for manual selection. In the backend, the portal computes CAMI benchmarking metrics using MetaQUAST [7] for assembly, AMBER [8] for binning, and OPAL [9] for profiling. Users can then select evaluation parameters of each tool and initiate the evaluation by clicking on the Evaluate button. As output, the portal displays the performance metrics and visualizations employed in the CAMI challenges. Results are visible only to the user via a submission code. Optionally, users can log in to provide metadata for reproducibility of the tool result submission, rank the results, and make them accessible to all portal visitors.

#### Software implementation

The CAMI Benchmarking Portal is implemented using the Python web framework Django. To handle the execution of a pool of evaluation requests, Django Q queues and manages the requests. It forwards commands to the Slurm Workload Manager on a separate large compute system, which runs MetaQUAST, AMBER, or OPAL, depending on the file type. A PostgreSQL database records users, sessions, evaluation requests and parameters, computed metrics, and metadata of user-uploaded files, such as software version and parameters used. It also stores information of the CAMI datasets and samples, which can be updated, for example, when CAMI creates a new benchmark dataset for use on the portal. All visualizations are interactive and created using the Bokeh Python library.

#### CAMI datasets

CAMI benchmark datasets representing realistic metagenomic data were created for the two challenge editions, CAMI I and II (Table 1). These datasets not only offered insights into the performance of computational methods, but also encouraged participation of the research community in establishing best practices, evaluation procedures, quality metrics, and standardized file formats in the field. Furthermore, they have become an important source of benchmarking data for numerous independent studies [12, 13].

The so-called “toy” datasets, simulated from public genomes and made available months before the challenge period, allowed participants to test their methods and familiarize themselves with the challenges. The challenge datasets were simulated exclusively from newly sequenced genomes or combined with public ones. To allow researchers to find methods suited for their needs, the datasets also vary in other properties, such as the number of samples, genomes, strains, microbial community environments, and simulated sequencing technology (https://cami-challenge.org/datasets/).

## Results

**Figure 1.**
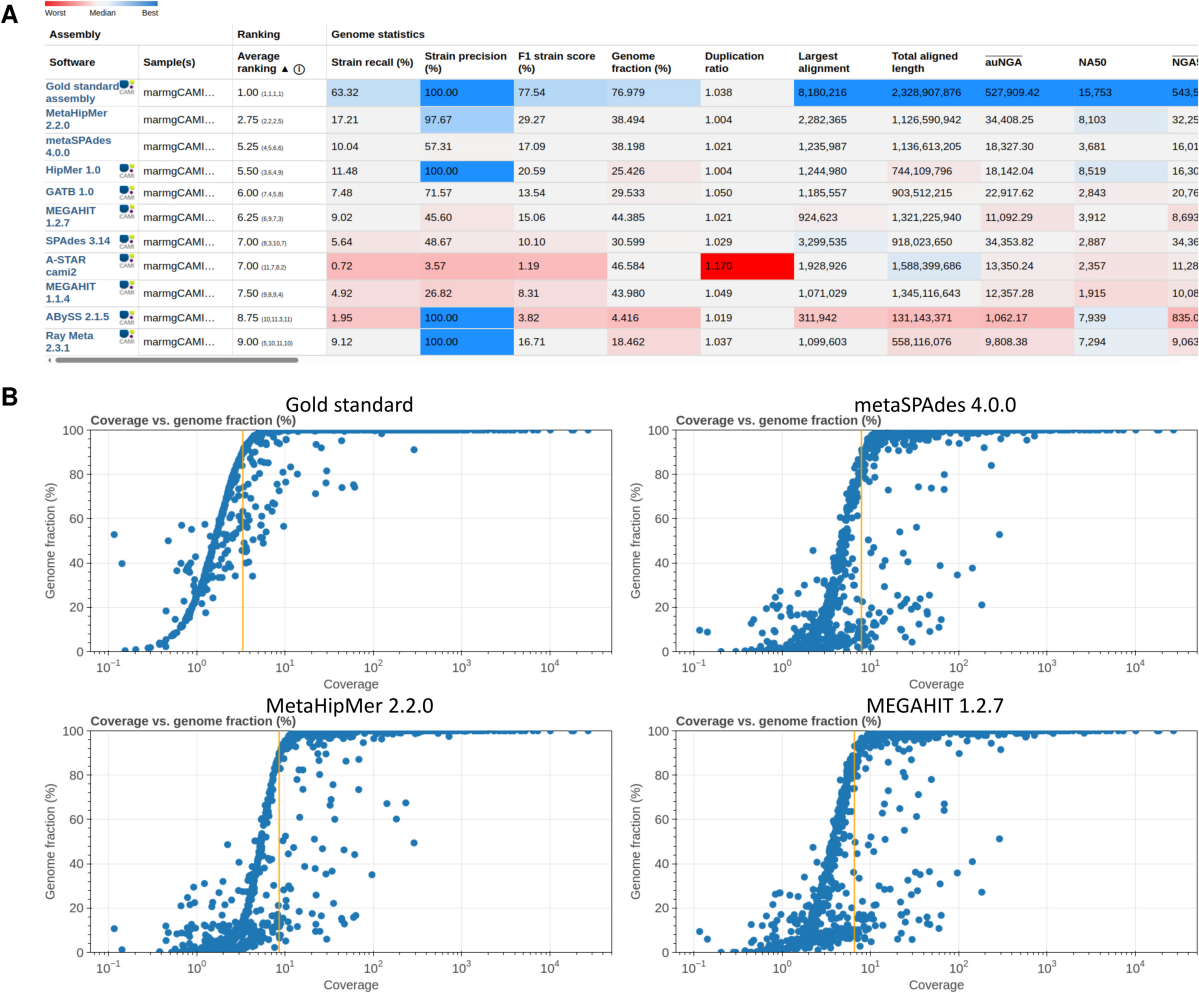
Assembly results from the CAMI Benchmarking Portal for the CAMI II marine dataset. (**A**) Heatmap table of public results. Blue indicates better performance and red indicates worse. Assemblies are assessed with MetaQUAST using the underlying genomes and ranked with a combination of metrics (see main text). The table is sortable by all metrics and horizontally scrollable to display additional metrics. (**B**) Scatter plots of genome coverage (*x*-axis) and assembled genome fraction (*y*-axis). The orange vertical line denotes the minimum coverage at which a genome is assembled with ≥90% genome fraction.

We illustrate the evaluation of metagenome assembly, genome and taxonomic binning, and taxonomic profiling using the marine Illumina HiSeq benchmark dataset of the CAMI II challenge. Our benchmarking includes top-performing software from the challenge as well as updated versions of popular and relevant tools. For all evaluation categories, results that the user set as public are ranked and presented in a heatmap-like table of the corresponding dataset and samples with category-specific metrics, where blue indicates better performance and red indicates worse (Fig. 1A and Supplementary Figs. S1, S4, S6, and S7). Rankings are computed across multiple metrics and averaged for a performance overview, currently following the ranking approach of the CAMI II challenge while incorporating community-suggested updates to effectively reflect methodological progress. The table is sortable by each metric on the portal by clicking on the respective metric. Results from the CAMI II challenge are marked with a CAMI symbol in the first column, while newly generated results are unmarked. Private results are not ranked and shown only for the submitter on the page of the specific submission. Notably, all underlying dataset genomes and gold standards representing theoretically optimal solutions became accessible to users after the conclusion of the challenges (https://cami-challenge.org/schedule/).

### Assembly

We evaluated eight metagenome assemblies—each a co-assembly of the 10 marine samples from the CAMI II challenge—alongside the gold standard assembly and assemblies of two recent software versions, MetaHipMer 2.2.0 [14] and metaSPAdes 4.0.0 [15]. The gold standard assembly represents an error-free assembly formed by the contiguous genome positions covered by the simulated sequence reads. To compute reference-based metrics, the CAMI Benchmarking Portal applies MetaQUAST using the 977 underlying genomes of the marine dataset (Fig. 1A). Additional metrics are calculated for measuring the strain resolution of the assemblies in the CAMI II challenge. Strain recall is the proportion of genomes that are assembled with ≥90% completeness and ≤5 mismatches per 100 kb, relative to the total number of genomes. Strain precision is the proportion of assembled genomes that meet the same criteria (≥90% completeness and ≤5 mismatches per 100 kb), relative to the number of genomes assembled with ≥90% completeness. Furthermore, we define sequence identity (IDY) as 100 minus the number of Ns, mismatches, and indels per 100 bp.

Assemblies are ranked overall based on the F1 score of strain recall and precision, the area under the NGAx curve (auNGA), IDY, and the genome fraction. This metric set updates the CAMI II challenge ranking [5]. In particular, auNGA replaces NGA50, as a more robust metric of average genome-wide assembly contiguity and contig-level correctness (https://lh3.github.io/2020/04/08/a-new-metric-on-assembly-contiguity). IDY, derived from three mismatch metrics as a higher-level measure of nucleotide correctness, replaces the previously used “number of misassemblies” in the ranking. Strain recall and precision are summarized in the F1 score, reflecting the overall correctness of strain-level genome recovery. Among the evaluated methods, MetaHipMer 2.2.0 ranked best across these metrics, followed by metaSPAdes 4.0.0, HipMer 1.0, GATB 1.0 [16], and MEGAHIT 1.2.7 [17].

Genome coverage affects assembly quality [5, 6] and is plotted versus the assembled genome fraction for each submitted result (examples in Fig. 1B). The gold standard assembly included genomes with ≥90% genome fraction starting at 3.3× coverage, MetaHipMer 2.2.0 at 8.6×, metaSPAdes 4.0.0 at 7.9×, and MEGAHIT 1.2.7 at 6.6×.

### Genome binning

**Figure 2.**
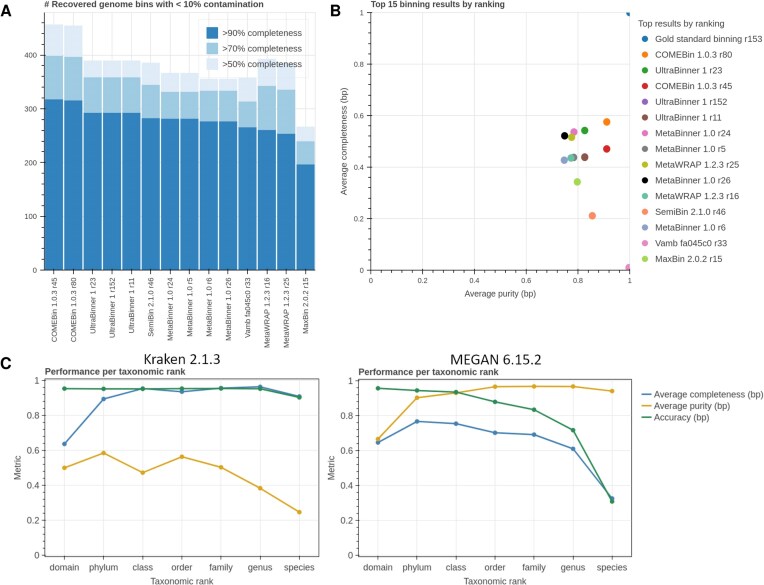
Genome (A and B) and taxonomic binning results (C) from the CAMI Benchmarking Portal for the CAMI II marine dataset. (**A**) Number of genomes recovered with <10% contamination and >50%, >70%, and >90% completeness, denoting low- to high-quality recovered genomes. (**B**) Scatter plot of average purity (*x*-axis) and average completeness in base pairs (*y*-axis). (**C**) Average completeness, average purity, and accuracy in base pairs (*y*-axis) from domain to species (*x*-axis) for taxonomic assignments of the gold standard assembly by Kraken 2.1.3 and MEGAN 6.15.2. For additional taxonomic binning results, see Supplementary Figs S3 and S4 or visit the CAMI Benchmarking Portal.

Genome binning allows for the recovery of genomes from metagenomic data by grouping sequences that belong to the same genome, usually from a metagenome assembly. We evaluated the performance of genome binners from the CAMI II challenge on the gold standard assembly of the marine dataset, along with the recently released binners COMEBin 1.0.3 [18] and SemiBin 2.1.0 [19]. Similarly, as for assembly, metrics computed with AMBER are included in a heatmap (Supplementary Fig. S1) and several plots (Fig. 2A and B). COMEBin recovered the highest number of high-quality genomes (318), followed by UltraBinner (293) and MetaBinner [20] (282, Fig. 2A). COMEBin also performed best in terms of F1 score reflective of both the average purity and completeness (70.5%), whereas Vamb [21] had the highest purity (99.7%, Fig. 2B). Currently, the CAMI Benchmarking Portal ranks genome binnings overall by average completeness and purity, adjusted Rand index, percentage of binned base pairs, and number of recovered genomes with >90% completeness and <10% contamination. The inclusion of the number of recovered genomes as a ranking metric updates the CAMI II ranking, as it is also a widely used metric [18–20]. The top three methods ranked across these metrics were the same that recovered the most high-quality genomes.

We also evaluated the performance of several methods on data including or excluding plasmids and other high-copy circular elements. In the latter case, completeness was higher by 8–10 percentage points for most methods, e.g. 57.5% compared to 47.1% for COMEBin and 42.6% compared to 34.5% for MaxBin 2.2.7 [22] (Supplementary Fig. S1), confirming that such elements are more difficult for binners to recover [23]. The portal also provides a view of the metrics per bin for every evaluation, allowing for a detailed investigation of the results (Supplementary Fig. S2).

### Taxonomic binning

Taxonomic binners assign sequence reads or assembled contigs a taxonomic label. Taxonomic assignments at a low rank, such as species, also imply assignments at higher ranks up to the domain level according to the NCBI taxonomy. We evaluated 56 results on the marine dataset gold standard assembly and 56 on short reads from 9 different methods and versions and the gold standard binning. The results of Metabuli 1.0.8 [24] and Kraken 2.1.3 [25] were newly added to the available CAMI II results on the portal. Currently, the CAMI Benchmarking Portal ranks the method results at each taxonomic level following the CAMI II consensus based on the average completeness and purity, the accuracy in base pairs, and the F1 score, and averages over the ranks for the individual metrics. Visualizations of the first three metrics are available for every result at each taxonomic rank (Fig. 2C and Supplementary Fig. S3). Similarly, as for genome binning, the portal provides a detailed view of the metrics per bin for every evaluation (Supplementary Fig. S5). On gold standard assemblies, Kraken 2.1.3 and MEGAN 6.15.2 [26] performed best overall across taxonomic levels (Supplementary Fig. S3). At the species level, Kraken 2.1.3 performed best, followed by Kraken 2.0.8 beta and MEGAN 6.15.2. On the short reads, Kraken 2.0.8 beta performed best, followed by Metabuli 1.0.8 [24] and NBC++ [27] at the species level, and Ganon 0.1.4 [28] and Kraken 2.0.8 beta showed the best performance overall across taxonomic levels.

### Taxonomic profiling

**Figure 3.**
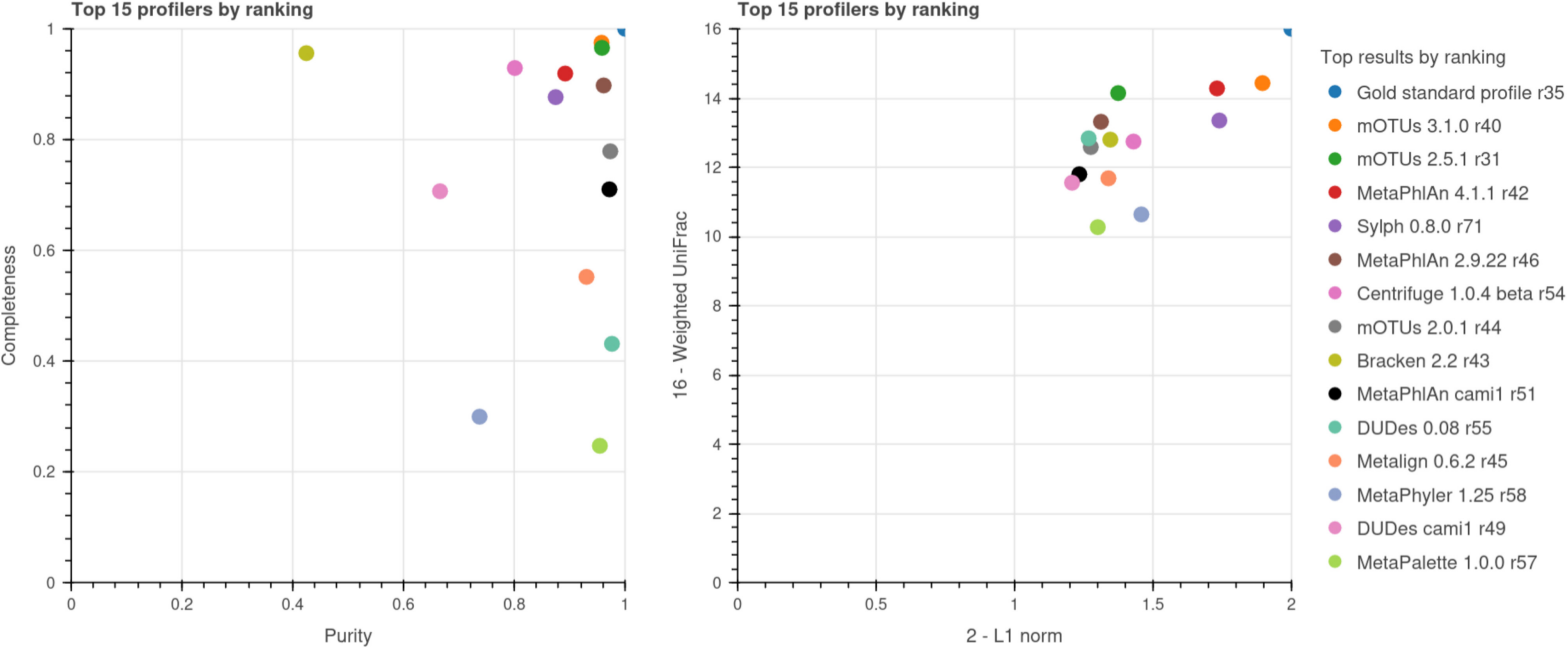
Taxonomic profiling results from the CAMI Benchmarking Portal for the CAMI II marine dataset at the genus level. The left panel shows purity (*x*-axis) versus completeness (*y*-axis), while the right panel shows 2 − L1 norm (*x*-axis) versus 16 − weighted UniFrac (*y*-axis). Values are averages across the 10 dataset samples. Results for individual samples and all taxonomic ranks, from domain to species and strain, are also available on the portal.

Taxonomic profilers provide insights into the composition of the microbial communities from sequence read samples by determining the presence and relative abundances of taxa across taxonomic ranks, without necessarily labeling each sequence individually. The portal currently includes 184 results for the marine dataset from 22 profiling methods and versions and the gold standard profile, from domain to strain level. mOTUs 3.1.0 [29], MetaPhlAn 4.1.1 [30], and Sylph 0.8.0 [12] are among the more recent methods included in addition to the CAMI II results. At the genus level, mOTUs 3.1.0 and 2.5.1 achieved the highest completeness (97.5% and 96.5%) and F1 score of completeness and purity (96.6% and 96.2%), and DUDes 0.08 [31] and mOTUs 2.0.1 had the highest purity (97.6% and 97.3%; Fig. 3 and Supplementary Fig. S6). This highlights their strengths in detecting the presence or absence of taxa. mOTUs 3.1.0 and MetaPhlAn 4.1.1 had the lowest weighted UniFrac error (1.5 and 1.7). mOTUs 3.1.0 also had the lowest L1 norm error (0.10), followed by Sylph 0.8.0 (0.26), indicating their strong performance in predicting microbial abundances at the genus level.

The CAMI Benchmarking Portal also provides an overall ranking of profiles at each taxonomic level, which currently following the CAMI II consensus is created by summarizing the individual rankings for completeness, purity, F1 score, L1 norm error, Bray–Curtis distance, and absolute difference of the Shannon equitability to the gold standard, as well as the weighted UniFrac error. mOTUs 3.1.0 and 2.5.1, and MetaPhlAn 4.1.1 ranked highest across these metrics at the genus level (Supplementary Fig. S6) and on average from domain to species.

Evaluation at the strain level is complicated by the lack of standard NCBI taxonomic identification codes. However, the number of detected taxa and their abundance evenness, measured with the Shannon equitability index, should reflect the alpha diversity of the gold standard profile. In this analysis, Sylph 0.8.0 and Metalign 0.6.2 [32] stood out, achieving Shannon equitability indices 0.768 and 0.753, respectively. Both closely matched the gold standard value of 0.778 (Supplementary Fig. S7). Metalign 0.6.2 also detected 457 strains, the closest to the 635 strains in the gold standard profile. For every evaluation and taxonomic level, the portal also shows a detailed view of the results in terms of true and false positives and false negatives (Supplementary Fig. S8).

## Conclusions

Benchmarking computational metagenomic methods using standardized, unbiased procedures, metrics, and benchmark datasets is essential for creating a fair and accurate snapshot of the state of the start for this rapidly evolving and growing research field. This helps researchers find the most appropriate and up-to-date methods for their studies. CAMI, the community-driven initiative for the Critical Assessment of Metagenome Interpretation, has established such principles and best practices in agreement with the community's suggestions and needs, through extensive collaboration in workshops, conferences, and benchmarking challenges [5, 6].

The CAMI portal (https://cami-challenge.org/) has since 2015 been the starting point for users to find all CAMI resources, such as benchmark datasets, articles, and guidelines, challenge information, file format descriptions, and tutorials. While already some solutions for online evaluations exist, such as for users to assess their single microbial genome assemblies [33], or metagenome binning and profiling results on user-provided gold standards [34], benchmarking using the combined CAMI resources, as suggested in [35], as well as comparisons to other tool results, including rankings, needed to be carried out offline with technical expertise and substantial time investments.

Therefore, to facilitate continuous and accessible benchmarking, we developed the CAMI Benchmarking Portal, which is an easy-to-use web platform integrating the evaluation tools MetaQUAST [7], AMBER [8], and OPAL [9] for assessing metagenome assembly, genome and taxonomic binning, and taxonomic profiling. The portal allows users to evaluate their results on CAMI datasets, visualize them in interactive plots and heatmaps, and compare and rank them against the results of other users, as well as the results from past CAMI challenges. Publicly shared results remain accessible to all visitors of the portal, making it a valuable repository for benchmarking reference. Reproducibility is enabled through designated metadata fields, such as information on software version, parameters, and reference datasets used.

We demonstrate the portal’s capabilities by reproducing previous CAMI challenge results, incorporating recent software, and ranking them using the community-defined evaluation criteria from prior challenges, while at the same time including updated community-defined evaluation criteria to more effectively reflect methodological progress. For instance, the current assembly ranking now incorporates sequence identity (IDY) and auNGA, a more robust metric than NGA50, while the genome binning ranking accounts for the number of recovered high-quality genome bins. Performance improvements across all assessed software categories highlight ongoing advancements. Notably, new results on data of previous challenges must be interpreted with caution, as the genomes underlying the benchmark datasets have since been made public and incorporated into reference databases used frequently by taxonomic profiling and binning methods, unlike at the time of the challenges. Furthermore, the provision of the ground truths after the challenges allows for consecutive optimization rounds directly on these datasets and performance improvements, different from results submitted to an ongoing challenge. This underscores the importance of blind benchmarks such as the CAMI challenges, in which participants do not know the correct answers in advance and need to deal with new data, as in a real-world scenario. The portal provides a hybrid solution of blind and continuous benchmarking, making it a powerful tool for iterative method development and performance evaluation both during and between CAMI challenges. In the future, the portal will provide new benchmark datasets and host new challenges, as well as support additional method categories, such as for pathogen detection.

## Supplementary Material

gkaf369_Supplemental_File

## Data Availability

The CAMI Benchmarking Portal is freely available at https://cami-challenge.org/. Results of metagenome assembly, genome and taxonomic binning, and taxonomic profiling can be uploaded and evaluated at https://cami-challenge.org/submit/. Evaluations and rankings of public results for the respective method category on various CAMI datasets are available at https://cami-challenge.org/assembly/, https://cami-challenge.org/genome_binning/, https://cami-challenge.org/taxonomic_binning/, and https://cami-challenge.org/taxonomic_profiling/. CAMI datasets and their DOIs are listed in Table 1 and at https://cami-challenge.org/datasets/.
