# Supplementary material for: CAMI Benchmarking Portal: online evaluation and ranking of metagenomic software
Source: Nucleic Acids Res. 2025 May 7;53(W1):W102–9. doi: 10.1093/nar/gkaf369 (PMC12230735; doi:10.1093/nar/gkaf369)
Supplement: gkaf369_Supplemental_File [file gkaf369_supplemental_file.pdf]

Supplementary Figures for

CAMI Benchmarking Portal: online evaluation and ranking of metagenomic software

Fernando Meyer[1,2,3], Gary Robertson[1,2,3], Zhi-Luo Deng[1,2,3], David Koslicki[4], Alexey Gurevich[5,6] and Alice C. McHardy[1,2,3,7,8]*

[1] Computational Biology of Infection Research, Helmholtz Centre for Infection Research (HZI), Braunschweig, Germany.
[2] Braunschweig Integrated Centre of Systems Biology (BRICS), Technische Universität Braunschweig, Braunschweig, Germany.
[3] Initiative for the Critical Assessment of Metagenome Interpretation (CAMI).
[4] Penn State University, University Park, PA, USA.
[5] Helmholtz Institute for Pharmaceutical Research Saarland (HIPS), Helmholtz Centre for Infection Research (HZI), Saarbrücken, Germany.
[6] Center for Bioinformatics Saar and Saarland University, Saarland Informatics Campus, Saarbrücken, Germany.
[7] German Center for Infection Research (DZIF), Hannover-Braunschweig site, Germany.
[8] Cluster of Excellence RESIST (EXC 2155), Hannover Medical School, Hannover, Germany.

* To whom correspondence should be addressed. Tel: +49 531 391 55271; Email: alice.mchardy@helmholtz-hzi.de

Worst Median Best

Genome binning	Metrics per bin	Ranking	Quality of bins: all bins have the same weight						Quality for sample		
**Software**	**# bins**	**Average ranking ▲ ⓘ**	**Average purity (bp)**	**Average purity (seq)**	**Average completeness (bp)**	**Average completeness (seq)**	**F1 score (bp)**	**F1 score (seq)**	**Accuracy (bp)**	**Accuracy (seq)**	**Misclassification rate (bp)**
Gold standard binning	976	1.00 (1,1,1,1,1)	1.000	1.000	1.000	1.000	1.000	1.000	1.000	1.000	0.000
COMEBin 1.0.3	682	5.80 (2,5,6,13,3)	0.911	0.837	0.575	0.480	0.705	0.610	0.694	0.160	0.108
UltraBinner 1	596	6.60 (3,8,3,15,4)	0.826	0.527	0.542	0.448	0.655	0.484	0.634	0.116	0.114
COMEBin 1.0.3	685	6.80 (7,6,6,13,2)	0.911	0.837	0.471	0.393	0.621	0.535	0.692	0.160	0.108
UltraBinner 1	596	7.60 (8,8,3,15,4)	0.826	0.527	0.440	0.364	0.574	0.430	0.632	0.115	0.114
UltraBinner 1	596	7.80 (9,8,3,15,4)	0.826	0.527	0.438	0.362	0.572	0.429	0.632	0.115	0.114
MetaBinner 1.0	652	8.20 (4,12,13,4,8)	0.784	0.456	0.536	0.448	0.637	0.452	0.658	0.123	0.183
MetaBinner 1.0	653	9.60 (10,13,13,4,8)	0.784	0.455	0.438	0.365	0.562	0.405	0.657	0.123	0.183
MetaWRAP 1.2.3	703	10.80 (6,14,18,2,14)	0.776	0.730	0.516	0.416	0.620	0.530	0.637	0.220	0.294
MetaBinner 1.0	658	11.20 (5,22,15,4,10)	0.749	0.451	0.522	0.437	0.615	0.444	0.645	0.116	0.199
MetaWRAP 1.2.3	717	11.80 (11,15,18,2,13)	0.773	0.726	0.435	0.354	0.557	0.476	0.636	0.219	0.294
SemiBin 2.1.0	1764	12.00 (25,7,2,19,7)	0.855	0.792	0.211	0.173	0.339	0.284	0.606	0.063	0.096
MetaBinner 1.0	660	12.80 (12,23,15,4,10)	0.747	0.450	0.427	0.358	0.543	0.399	0.644	0.115	0.199
Vamb fa045c0	43393	15.40 (28,2,17,18,12)	0.997	0.996	0.010	0.008	0.020	0.016	0.621	0.089	0.116
MaxBin 2.0.2	516	15.60 (18,11,8,24,17)	0.797	0.496	0.343	0.296	0.479	0.371	0.511	0.083	0.177
MaxBin 2.2.7	515	15.80 (13,18,11,22,15)	0.769	0.485	0.426	0.369	0.548	0.419	0.514	0.083	0.179
MaxBin 2.0.2	516	16.60 (14,20,8,24,17)	0.764	0.481	0.423	0.365	0.544	0.415	0.512	0.083	0.177
MaxBin 2.2.7	515	16.60 (17,18,11,22,15)	0.769	0.485	0.345	0.299	0.477	0.370	0.512	0.083	0.179
MaxBin 2.0.2	516	17.40 (18,20,8,24,17)	0.764	0.481	0.343	0.296	0.473	0.367	0.511	0.083	0.177
CONCOCT 1.1.0	371	17.60 (15,24,23,4,22)	0.697	0.597	0.358	0.292	0.473	0.392	0.432	0.118	0.463
CONCOCT 0.4.1	362	18.20 (16,26,20,4,25)	0.689	0.628	0.355	0.286	0.468	0.393	0.429	0.117	0.466
Autometa HGT 0.9.0	427	18.80 (24,3,27,20,20)	0.943	0.789	0.239	0.203	0.381	0.322	0.360	0.012	0.441
Autometa HGT 0.9.0	426	18.80 (22,4,27,20,21)	0.943	0.789	0.293	0.248	0.447	0.377	0.361	0.012	0.441
CONCOCT 0.4.1	373	19.20 (20,27,20,4,25)	0.678	0.612	0.295	0.239	0.411	0.344	0.429	0.117	0.467
CONCOCT 0.4.1	373	19.40 (21,28,20,4,24)	0.676	0.614	0.295	0.239	0.410	0.344	0.429	0.117	0.467
CONCOCT 1.1.0	373	19.40 (23,25,23,4,22)	0.696	0.596	0.289	0.236	0.408	0.338	0.431	0.118	0.464
MetaBAT 0.25.4	232	24.20 (26,16,25,27,27)	0.771	0.642	0.171	0.143	0.280	0.234	0.205	0.013	0.552
MetaBAT 0.25.4	232	24.40 (27,16,25,27,27)	0.771	0.642	0.138	0.115	0.234	0.195	0.204	0.013	0.552

Supplementary Figure S1. Genome binning results from the CAMI Benchmarking Portal for the gold standard assembly of the CAMI II marine dataset. Genome binnings are ranked based on the average completeness and purity in base pairs, adjusted Rand index, percentage of binned base pairs, and number of recovered genomes (>90% completeness and <10% contamination). The number of recovered genomes as a ranking metric was implemented in the portal, in addition to the other metrics already previously considered for ranking in the CAMI II challenge. Blue indicates better performance and red indicates worse. The table is sortable by all metrics and horizontally scrollable to display additional metrics.

Genome binning

Submission: 6af7be236eab47f7bda3

Evaluation: #80

Dataset: Marine

Sample: marmgCAMI2_short_read_pooled_gold_standard_assembly

Software: COMEBin 1.0.3

[Page 1 of 7] next last »

Bin ID	Most abundant genome	Purity (bp)	Completeness (bp)	Bin size (bp) ▼	True positives (bp)	True size of most abundant genome (bp)	Purity (seq)	Completeness (seq)	Bin size (seq)	True positives (seq)	True size of most abundant genome (seq)	Average seq length
115286	Otu405.0	0.201	0.907	15,854,695	3,187,858	3,515,402	0.001	0.200	1,745	1	5	9,085.8
34181	Otu128	0.262	0.781	15,373,713	4,025,496	5,156,583	0.076	0.762	1,058	80	105	14,530.9
137695	Otu289	0.292	0.931	14,606,673	4,261,286	4,579,336	0.034	0.541	1,581	53	98	9,238.9
71591	Otu395	0.360	0.946	12,756,593	4,592,272	4,853,417	0.308	0.792	2,082	641	809	6,127.1
163308	Otu796	0.481	0.872	11,644,932	5,601,035	6,421,151	0.127	0.726	417	53	73	27,925.5
114501	Otu659	0.276	0.559	10,400,695	2,871,313	5,131,947	0.046	0.414	785	36	87	13,249.3
2376	Otu1529	0.452	0.998	9,580,805	4,332,209	4,341,207	0.122	0.975	1,580	193	198	6,063.8
85124	Otu987	1.000	0.997	9,302,959	9,302,959	9,329,432	1.000	0.935	489	489	523	19,024.5
31054	Otu54	0.379	0.998	9,141,646	3,461,409	3,466,651	0.125	0.995	1,586	198	199	5,764.0
193012	Otu404.0	0.516	1.000	8,839,563	4,559,160	4,559,160	0.500	1.000	28	14	14	315,698.7
221465	Otu1358	1.000	0.987	8,813,785	8,813,785	8,928,984	1.000	0.863	1,313	1,313	1,522	6,712.7
19802	Otu1519	0.550	0.891	8,802,570	4,842,151	5,433,103	0.431	0.666	2,451	1,056	1,586	3,591.4
214838	Otu282.1	0.558	0.999	8,790,551	4,906,457	4,910,232	0.035	0.934	1,629	57	61	5,396.3
70523	Otu1057	0.189	0.320	8,545,568	1,618,766	5,050,756	0.203	0.247	1,068	217	878	8,001.5
162614	Otu198.0	0.537	0.972	8,295,703	4,451,790	4,580,430	0.327	0.917	168	55	60	49,379.2
776	Otu2290.0	0.322	0.996	8,237,647	2,655,528	2,667,193	0.076	0.946	1,600	122	129	5,148.5
142870	Otu968.0	0.513	0.863	8,215,921	4,217,775	4,890,170	0.912	0.727	647	590	812	12,698.5

Supplementary Figure S2. Detailed view from the CAMI Benchmarking Portal of the metrics per genome bin recovered by COMEBin 1.0.3 from the gold standard assembly of the CAMI II marine dataset. COMEBin recovered 682 bins. Exemplarily shown are the metrics for the largest ones in base pairs of the contigs in the bins.

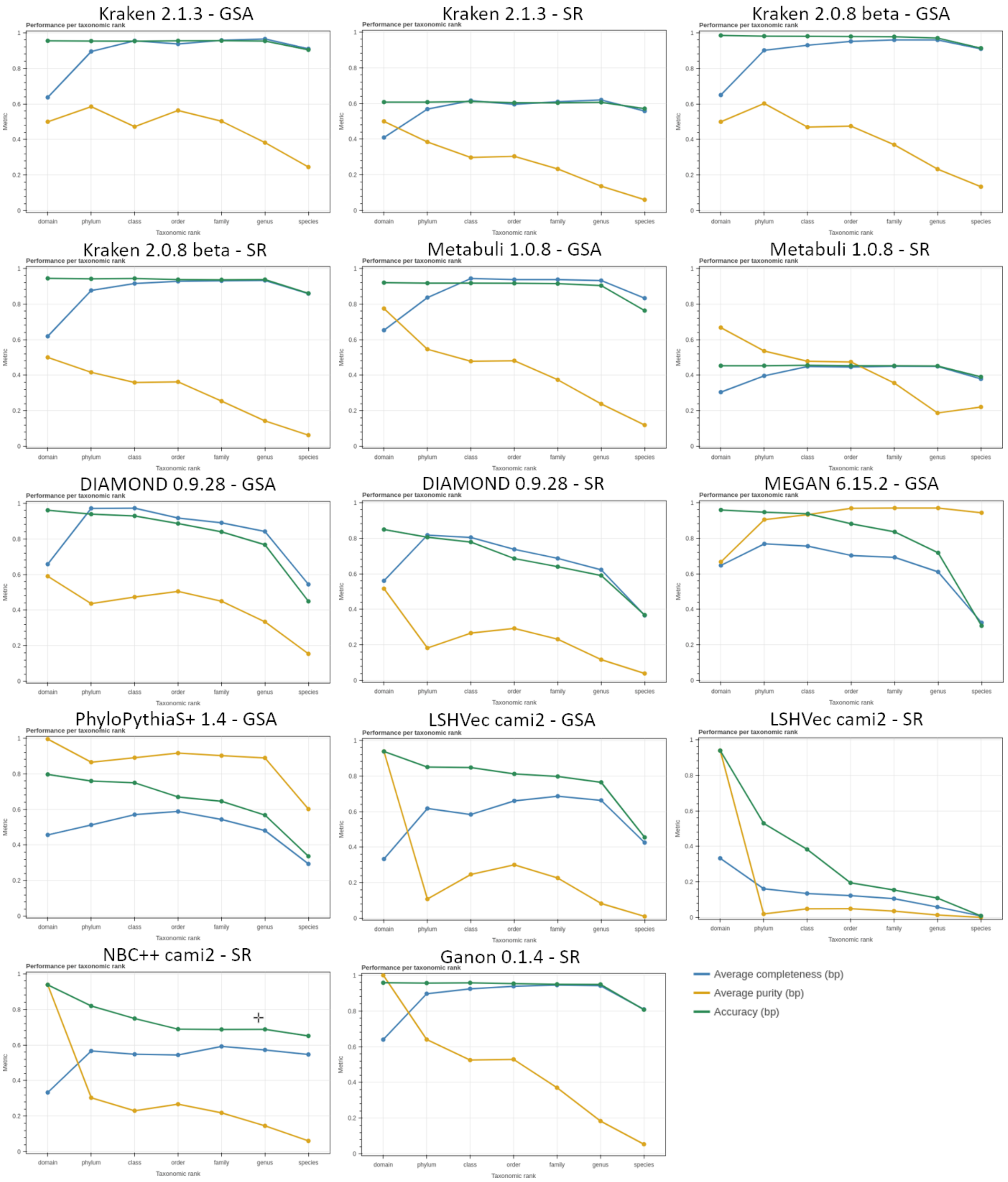

Supplementary Figure S3. Taxonomic binning performances in terms of average completeness and purity, and accuracy in base pairs (y-axis) from the CAMI Benchmarking Portal for the CAMI II marine dataset. Results are assessed across taxonomic ranks, from domain to species (x-axis). GSA stands for binning of the gold standard assembly and SR for binning of the dataset short reads. Metrics for SR are averaged over the 10 samples of the dataset.

Worst Median Best

Taxonomic binning of the gold standard assembly (species level)

Taxonomic binning	Metrics per bin	Ranking	Quality of bins: all bins have the same weight						Quality for sample		
Software	# bins	Average ranking ▲ ⓘ	Average purity (bp)	Average purity (seq)	Average completeness (bp)	Average completeness (seq)	F1 score (bp)	F1 score (seq)	Accuracy (bp)	Accuracy (seq)	Misclassification rate (bp)
Gold standard binning	478	1.00 (1,1,1,1)	1.000	1.000	1.000	1.000	1.000	1.000	1.000	1.000	0.000
Kraken 2.1.3	1852	3.50 (3,4,4,3)	0.246	0.232	0.909	0.898	0.387	0.369	0.904	0.847	0.050
Kraken 2.0.8 beta	3306	4.00 (2,6,6,2)	0.135	0.116	0.909	0.902	0.235	0.206	0.913	0.852	0.059
MEGAN 6.15.2	491	4.75 (7,2,2,8)	0.942	0.933	0.325	0.302	0.483	0.457	0.308	0.326	0.032
DIAMOND 0.9.28	2935	5.25 (5,5,5,6)	0.154	0.146	0.544	0.530	0.240	0.229	0.449	0.313	0.026
PhyloPythiaS+ 1.4	587	5.25 (8,3,3,7)	0.602	0.528	0.294	0.231	0.395	0.321	0.337	0.041	0.297
Metabuli 1.0.8	3556	5.50 (4,7,7,4)	0.120	0.104	0.832	0.829	0.209	0.185	0.762	0.720	0.106
LSHVec cami2	32091	6.75 (6,8,8,5)	0.011	0.008	0.425	0.354	0.021	0.015	0.455	0.141	0.508

Taxonomic binning of short reads (species level)

Taxonomic binning		Ranking	Quality of bins: all bins have the same weight						Quality for sample		
Software	Samples	Average ranking ▲ ⓘ	Average purity (bp)	Average purity (seq)	Average completeness (bp)	Average completeness (seq)	F1 score (bp)	F1 score (seq)	Accuracy (bp)	Accuracy (seq)	Misclassification rate (bp)
Gold standard binning	marmgCAMI...	1.00 (1,1,1,1)	1.000	1.000	1.000	1.000	1.000	1.000	1.000	1.000	0.000
Kraken 2.0.8 beta	marmgCAMI...	2.50 (2,3,3,2)	0.063	0.063	0.858	0.858	0.118	0.118	0.860	0.860	0.041
Metabuli 1.0.8	marmgCAMI...	4.00 (6,2,2,6)	0.221	0.221	0.379	0.379	0.279	0.279	0.390	0.390	0.027
NBC++ cami2	marmgCAMI...	4.25 (5,4,4,4)	0.061	0.061	0.547	0.547	0.110	0.110	0.651	0.651	0.349
Ganon 0.1.4	marmgCAMI...	4.50 (3,6,6,3)	0.054	0.054	0.809	0.809	0.101	0.101	0.807	0.807	0.030
Kraken 2.1.3	marmgCAMI...	4.75 (4,5,5,5)	0.061	0.061	0.558	0.558	0.110	0.110	0.571	0.571	0.019
DIAMOND 0.9.28	marmgCAMI...	7.00 (7,7,7,7)	0.040	0.040	0.366	0.366	0.072	0.072	0.368	0.368	0.027
LSHVec cami2	marmgCAMI...	8.00 (8,8,8,8)	0.002	0.002	0.009	0.009	0.003	0.003	0.010	0.010	0.990

Supplementary Figure S4. Taxonomic binning results from the CAMI Benchmarking Portal at the species level for the CAMI II marine dataset. Top: binning results for the gold standard assembly. Bottom: binning results for the short reads, averaged across the 10 dataset samples. Taxonomic binnings are ranked at each taxonomic level based on the average completeness and purity, accuracy in base pairs, and F1 score. Blue indicates better performance and red indicates worse. The table is sortable by all metrics and horizontally scrollable to display additional metrics. Results per sample and for every taxonomic rank, from domain to species, are also available on the portal.

Taxonomic binning (phylum)

Submission: 0209a09b3a4a4ca6b67c

Evaluation: #163

Dataset: Marine

Sample: marmgCAMI2_short_read_pooled_gold_standard_assembly

Rank: domain phylum class order family genus species

Software: Kraken 2.1.3

Taxon ID	Scientific name	Purity (bp)	Completeness (bp)	Bin size (bp) ▼	True positives (bp)	True size (bp)	Purity (seq)	Completeness (seq)	Bin size (seq)	True positives (seq)	True size (seq)
1224	Proteobacteria	0.999	0.954	1,540,683,298	1,539,309,028	1,613,890,064	0.999	0.896	823,523	822,902	918,085
976	Bacteroidetes	0.999	0.956	281,817,334	281,659,310	294,520,372	0.999	0.899	106,541	106,451	118,464
201174	Actinobacteria	0.996	0.926	174,077,262	173,421,914	187,257,449	0.998	0.922	152,880	152,623	165,570
28890	Euryarchaeota	1.000	0.972	129,435,645	129,417,254	133,192,769	1.000	0.954	130,383	130,370	136,621
1239	Firmicutes	0.997	0.959	123,570,418	123,187,915	128,449,458	0.998	0.932	79,530	79,353	85,155
1117	Cyanobacteria	0.999	0.989	99,802,427	99,734,136	100,803,267	1.000	0.964	70,202	70,169	72,807
200918	Thermotogae	1.000	0.988	30,188,683	30,188,683	30,549,706	1.000	0.982	35,254	35,254	35,918
28889	Crenarchaeota	0.968	0.997	15,514,687	15,014,784	15,055,611	1.000	0.989	10,522	10,518	10,637
1090	Chlorobi	0.999	1.000	9,911,702	9,901,757	9,904,930	0.999	0.997	4,464	4,461	4,476
651137	Thaumarchaeota	1.000	0.732	9,168,391	9,167,310	12,531,268	1.000	0.907	4,337	4,335	4,779
200930	Deferribacteres	1.000	0.999	7,798,829	7,798,829	7,807,512	1.000	0.991	5,034	5,034	5,082
203691	Spirochaetes	0.997	1.000	6,362,294	6,342,719	6,342,719	0.583	1.000	24	14	14
1930617	Calditrichaeota	1.000	1.000	4,978,870	4,978,230	4,978,230	0.917	1.000	12	11	11
200783	Aquificae	1.000	1.000	4,919,435	4,918,431	4,920,831	1.000	0.995	3,319	3,318	3,334
1297	Deinococcus-Thermus	0.996	1.000	4,532,187	4,512,616	4,513,496	0.996	0.998	2,120	2,112	2,117
203682	Planctomycetes	0.990	1.000	3,803,443	3,765,539	3,767,029	0.990	0.996	1,714	1,697	1,704
32066	Fusobacteria	0.996	1.000	3,145,300	3,132,217	3,132,217	0.300	1.000	10	3	3
134625	Kiritimatiellaeota	1.000	0.999	2,626,555	2,626,555	2,628,650	1.000	0.996	2,938	2,938	2,949
508458	Synergistetes	0.995	1.000	2,010,203	1,999,613	1,999,613	0.667	1.000	3	2	2

Supplementary Figure S5. Detailed view from the CAMI Benchmarking Portal of the metrics per bin (i.e., each row in the table) and respective taxonomic label by Kraken 2.1.3 for the gold standard assembly of the CAMI II marine dataset, at the phylum level. Kraken computed 40 bins at this level. Exemplarily shown are the metrics for the largest ones in base pairs of the contigs in the bins.

Worst Median Best

Taxonomic profiling		Ranking	Alpha diversity		Presence/absence of taxa						
Software	Samples	Average ranking ▲ ⓘ	Shannon diversity	Shannon equitability	Completeness	Purity	F1 score	True positives	False positives	False negatives	Jaccard index
Gold standard profile	marmgCAMI…	1.00 (1,1,1,1,1,1,1)	1.838	0.347	1.000	1.000	1.000	200	0	0	1.000
mOTUs 3.1.0	marmgCAMI…	5.00 (2,7,2,2,2,18,2)	4.037	0.759	0.975	0.957	0.966	195	8	5	0.935
mOTUs 2.5.1	marmgCAMI…	6.14 (3,6,3,7,6,14,4)	3.922	0.739	0.965	0.958	0.962	193	8	6	0.927
MetaPhlAn 4.1.1	marmgCAMI…	6.71 (6,10,5,4,4,15,3)	3.992	0.749	0.919	0.892	0.905	184	22	16	0.827
Sylph 0.8.0	marmgCAMI…	7.57 (8,11,6,3,3,17,5)	3.988	0.752	0.876	0.875	0.876	176	25	24	0.779
MetaPhlAn 2.9.22	marmgCAMI…	7.71 (7,5,4,10,9,13,6)	3.853	0.737	0.898	0.962	0.928	180	7	20	0.866
Centrifuge 1.0.4 beta	marmgCAMI…	7.86 (5,13,8,6,5,9,9)	3.778	0.693	0.929	0.801	0.860	186	46	14	0.755
mOTUs 2.0.1	marmgCAMI…	9.14 (10,3,7,12,10,12,10)	3.729	0.735	0.779	0.973	0.865	156	4	44	0.762
Bracken 2.2	marmgCAMI…	9.29 (4,18,14,8,7,6,8)	4.123	0.674	0.956	0.424	0.586	191	264	8	0.416
MetaPhlAn cami1	marmgCAMI…	10.57 (11,4,9,15,13,11,11)	3.559	0.714	0.710	0.972	0.820	142	4	58	0.696
DUDes 0.08	marmgCAMI…	12.14 (18,2,13,14,11,20,7)	3.482	0.777	0.431	0.976	0.598	86	2	114	0.426
Metalign 0.6.2	marmgCAMI…	12.29 (16,9,11,9,8,21,12)	3.812	0.799	0.552	0.930	0.692	110	8	90	0.530
MetaPhyler 1.25	marmgCAMI…	13.43 (22,15,16,5,18,2,16)	1.921	0.437	0.299	0.737	0.425	59	21	140	0.270
DUDes cami1	marmgCAMI…	13.71 (12,16,12,18,15,10,13)	3.738	0.698	0.706	0.666	0.685	141	71	59	0.521
MetaPalette 1.0.0	marmgCAMI…	14.43 (23,8,18,11,19,4,18)	2.473	0.640	0.247	0.954	0.386	48	2	151	0.244
NBC++ cami2	marmgCAMI…	14.57 (9,22,22,17,14,3,15)	4.110	0.590	0.836	0.159	0.266	167	890	32	0.154
mOTUs cami1	marmgCAMI…	15.14 (13,14,10,21,21,8,19)	3.513	0.689	0.640	0.784	0.704	128	35	72	0.544
CCMetagen 1.1.3	marmgCAMI…	16.00 (20,12,15,16,16,16,17)	3.332	0.751	0.369	0.872	0.518	73	10	127	0.350
TIPP cami1	marmgCAMI…	16.29 (14,20,20,19,20,7,14)	4.217	0.682	0.591	0.245	0.346	118	366	82	0.209
FOCUS cami1	marmgCAMI…	17.43 (19,19,19,13,12,19,21)	4.192	0.774	0.410	0.365	0.386	82	143	118	0.239
TIPP 4.3.10	marmgCAMI…	18.00 (15,21,21,22,22,5,20)	4.053	0.654	0.567	0.231	0.328	113	378	87	0.196
FOCUS 1.5	marmgCAMI…	19.57 (21,17,17,20,17,23,22)	4.337	0.855	0.351	0.440	0.390	70	89	130	0.243
LSHVec cami2	marmgCAMI…	22.00 (17,23,23,23,23,22,23)	6.341	0.853	0.518	0.062	0.111	104	1,642	96	0.059

Supplementary Figure S6. Taxonomic profiling results from the CAMI Benchmarking Portal at the genus level for the CAMI II marine dataset. Metric values are averages across the 10 dataset samples. Blue indicates better performance and red indicates worse. Taxonomic profiles are ranked at each taxonomic level based on the completeness, purity, F1 score, L1 norm error, Bray-Curtis distance, Shannon equitability absolute difference to gold standard, and weighted UniFrac error. The table is sortable by all metrics and horizontally scrollable to display additional metrics. Results for individual samples and all taxonomic ranks, from domain to species and strain, are also available on the portal.

Worst Median Best

Taxonomic profiling		Ranking	Alpha diversity		Presence/absence of taxa						
Software	Samples	Average ranking ⓘ	Shannon diversity	Shannon equitability ▲	Completeness	Purity	F1 score	True positives	False positives	False negatives	Jaccard index
MetaPalette 1.0.0	marmgCAMI...	5.14 (2,2,2,2,2,8,18)	0.539	0.149	0.000	0.000		0	33	469	0.000
MetaPhlAn cami1	marmgCAMI...	4.29 (2,2,2,4,2,7,11)	2.482	0.496	0.000	0.000		0	149	469	0.000
FOCUS 1.5	marmgCAMI...	5.57 (2,2,2,3,2,6,22)	2.593	0.536	0.000	0.000		0	125	469	0.000
DUDes cami1	marmgCAMI...	4.57 (2,2,2,6,2,5,13)	3.343	0.572	0.000	0.000		0	344	469	0.000
FOCUS cami1	marmgCAMI...	5.43 (2,2,2,5,2,4,21)	3.977	0.692	0.000	0.000		0	315	469	0.000
Metalign 0.6.2	marmgCAMI...	4.29 (2,2,2,7,2,3,12)	4.610	0.753	0.000	0.000		0	457	469	0.000
Sylph 0.8.0	marmgCAMI...	3.29 (2,2,2,8,2,2,5)	3.919	0.768	0.000	0.000		0	166	469	0.000
Gold standard profile	marmgCAMI...	1.00 (1,1,1,1,1,1,1)	5.017	0.778	1.000	1.000	1.000	635	0	0	1.000

Supplementary Figure S7. Taxonomic profiling results from the CAMI Benchmarking Portal at the strain level for the CAMI II marine dataset, in ascending order of Shannon equitability. Metric values are averages across the 10 dataset samples. Completeness, purity, and other metrics have value zero due to a lack of standard NCBI taxonomic identification codes for strains. For the same reason, all identified taxa are evaluated as false positives in this example. However, the number of detected taxa and the Shannon equitability should reflect the alpha diversity of the gold standard profile. Taxonomic profiles are ranked at each taxonomic level by completeness, purity, F1 score, L1 norm error, Bray-Curtis distance, Shannon equitability absolute difference to gold standard, and weighted UniFrac error.

Taxonomic profiling (phylum)

Submission: 3a9bce7c5cf6418da87c

Evaluation: #40

Dataset: Marine

Sample: marmgCAMI2_short_read_sample_0

Rank: domain phylum class order family genus species strain

Software: mOTUs 3.1.0

Taxon ID	Relative abundance (%) ▼	True relative abundance (%)	Classification (TP=true positive, FP=false positive, FN=false negative)
1462430	-	0.171	FN
1224	69.174	69.049	TP
28890	7.282	6.691	TP
28889	6.875	6.777	TP
201174	4.526	4.670	TP
1239	3.010	2.990	TP
1117	2.774	2.705	TP
976	2.130	2.904	TP
1090	1.405	1.395	TP
1297	0.909	0.854	TP
651137	0.557	0.541	TP
200918	0.361	0.370	TP
192989	0.329	0.313	TP
32066	0.276	0.256	TP
200783	0.254	0.199	TP
544448	0.080	0.057	TP
203682	0.058	0.057	TP

Supplementary Figure S8. Detailed view from the CAMI Benchmarking Portal of the abundance profile exemplarily at the phylum level by mOTUs 3.1.0 for the CAMI II marine sample *marmgCAMI2_short_read_sample_0*. The table shows the method's computed and gold standard's true relative abundance per taxon for true positives (TP) and also indicates if the taxon is a false negative (FN) or false positive (FP, no case in this example). Relative abundances are normalized in this example. Such a table is available on the portal for every result and corresponding sample, at every taxonomic level.
